# *PGM2 *overexpression improves anaerobic galactose fermentation in *Saccharomyces cerevisiae*

**DOI:** 10.1186/1475-2859-9-40

**Published:** 2010-05-27

**Authors:** Rosa Garcia Sanchez, Bärbel Hahn-Hägerdal, Marie F Gorwa-Grauslund

**Affiliations:** 1 Department of Applied Microbiology, Lund University, P.O. Box 124, SE-22100 Lund, Sweden

## Abstract

**Background:**

In *Saccharomyces cerevisiae *galactose is initially metabolized through the Leloir pathway after which glucose 6-phosphate enters glycolysis. Galactose is controlled both by glucose repression and by galactose induction. The gene *PGM2 *encodes the last enzyme of the Leloir pathway, phosphoglucomutase 2 (Pgm2p), which catalyses the reversible conversion of glucose 1-phosphate to glucose 6-phosphate. Overexpression of *PGM2 *has previously been shown to enhance aerobic growth of *S. cerevisiae *in galactose medium.

**Results:**

In the present study we show that overexpression of *PGM2 *under control of the *HXT7'*promoter from an integrative plasmid increased the PGM activity 5 to 6 times, which significantly reduced the lag phase of glucose-pregrown cells in an anaerobic galactose culture. *PGM2 *overexpression also increased the anaerobic specific growth rate whereas ethanol production was less influenced. When *PGM2 *was overexpressed from a multicopy plasmid instead, the PGM activity increased almost 32 times. However, this increase of PGM activity did not further improve aerobic galactose fermentation as compared to the strain carrying *PGM2 *on the integrative plasmid.

**Conclusion:**

*PGM2 *overexpression in *S. cerevisiae *from an integrative plasmid is sufficient to reduce the lag phase and to enhance the growth rate in anaerobic galactose fermentation, which results in an overall decrease in fermentation duration. This observation is of particular importance for the future development of stable industrial strains with enhanced PGM activity.

## Background

Sustainable bioconversion of renewable lignocellulose raw material requires that all sugar components are efficiently utilized [1]. These raw materials are composed of a number of different monosaccharides one of which is galactose. It may constitute up to 18% of the total released monomeric sugar of pretreated softwood [2]. Actually the amount of galactose is higher than that of arabinose in certain raw materials such as willow [3] and spruce [4]. Galactose also makes up a substantial amount of other industrial media raw materials as beet molasses [5] and cheese whey [6].

Baker's yeast, which is the preferred organism for industrial ethanolic fermentation [7], catabolizes galactose via the Leloir pathway [8]. Galactose catabolism to the glycolytic intermediate glucose 6-phosphate is a complex sequence of reactions including phosphorylation and group transfer. Galactose is consumed and metabolized at a significantly lower rate than glucose since it delays induction of glycolytic genes [9]. In addition the Leloir pathway is controlled by glucose repression even in the presence of galactose [10].

When the structural genes for the galactose metabolizing enzymes were overexpressed, growth on galactose was impaired [11]. However increased galactose utilization and ethanol production was observed when genes encoding the galactose regulatory network were engineered [12]. A transcriptome analysis of two of these engineered *S. cerevisiae *strains, SO7 (p*GAL4, 2 μ*) and SO16 (Δ*gal6 *Δ*gal80 *Δ*mig1*), later identified a slight up-regulation of *PGM2 (GAL5) *to be a common denominator [13]. It was also verified that overexpression of *PGM2 *in *S. cerevisiae *improved galactose uptake under aerobic conditions while anaerobic conditions were not investigated [13].

The objective of the current study was to investigate the influence of overexpressing *PGM2 *in *S. cerevisiae *under anaerobic conditions. *PGM2 *was expressed both from a multicopy plasmid and from an integrative plasmid. The PGM activity increased 5 to 6-fold in galactose-grown cells when the gene was expressed from the integrative plasmid. *PGM2 *overexpression reduced the lag phase of glucose pre-grown cells and increased the maximum specific growth rate on galactose. The additional increase of PGM activity in strains expressing *PGM2 *from a multicopy plasmid did not further increase growth on galactose.

## Results

### Effect of *PGM2 *gene dosage

Two strains with different copy numbers of the gene *PGM2 *were constructed in the same genetic background (Table 1). In both strains, *PGM2 *was expressed under the control of the constitutive promoter *HXT7' *[14]. One strain expressed at least one additional integrated copy of *PGM2 *and was named **PGM2 i**. Its corresponding control strain was **Control i**. The other constructed strain overexpressed *PGM2 *from a multicopy plasmid and was named **PGM2 m**. Its control strain **Control m **carried the same plasmid without the structural gene.

**Table 1:** Plasmids and *S. cerevisiae *strains used in this study

	Relevant genotype/phenotype	Reference
		

**Plasmids**		

**YEplacHXT**	YEplac195, *HXT7p-PGKt URA3*	[27]

**YEplacHXT-PGM2**	YEplac195, *HXT7p-PGM2-PGKt URA3*	This work

**YDp-H**	*HIS3*	[33]

**YIplac211**	*URA3*	[30]

**YIplac211 HXT-PGM2**	Ylpac211, *HXT7p-PGM2-PGKt URA3*	This work

		

*S. cerevisiae *strains (*)		

**CEN.PK 113-11C**	*MAT***a ***his3Δ1 ura 3-52 MAL2-8c SUC2*	[28]

**TMB 3126**	CEN.PK 113-11C, *his3Δ1 *YEplacHXT *URA3*	This work

**TMB 3400**	genomic *S. cerevisiae *DNA (used as template to amplify *PGM2*)	[29]

**TMB 3127**	CEN.PK 113-11C, *his3Δ1 *YEplacHXT-PGM2 *URA3*	This work

**TMB 3128 "Control m"**	TMB 3126 *his3::HIS3 *YEplacHXT *URA3*	This work

**TMB 3129 "PGM2 m"**	TMB 3127 *his3::HIS3 *YEplacHXT-PGM2 *URA3*	This work

**TMB 3134**	CEN.PK 113-11C, *his3::HIS3 ura3*	This work

**TMB 3135 "Control i"**	CEN.PK 113-11C, *his3::HIS3 ura3::URA3*	This work

**TMB 3136 "PGM2 i"**	CEN.PK 113-11C, *his3::HIS3 ura3::URA3 HXT7'p-PGM2-PGKt*	This work

(*) Abbreviations of the most important genetic features are shown in quotation marks

The effect of the *PGM2 *copy number on the specific PGM activity was assessed in crude extracts of cells grown on galactose (Table 2). Strains **Control m **and **Control i **displayed the same specific PGM activity, 0.33 and 0.34 U mg^-1 ^protein, respectively, whereas the specific activity in the **PGM2 m **and **PGM2 i **strains increased to 10.40 and 1.81 U mg^-1 ^protein, respectively. Whereas PGM activity increased almost 32 fold in the multicopy strain, only a 17 fold increase was observed in the previously reported overexpression of *PGM2 *on multicopy vector [13] (Table 2). The difference may be ascribed to different strain background, different multicopy vector and different promoter.

**Table 2:** Specific PGM activity (U mg protein^-1^) and maximum specific growth rates on galactose

Strain	PGM activity (U mg protein^-1^)	Maximum specific growth rates (h^-1^)
**Control m**	0.33 ± 0.03	0.22 ± 0.01

**PGM2 m**	10.40 ± 1.58	0.30 ± 0.02

**Control i**	0.34 ± 0.01	0.24 ± 0.02

**PGM2 i**	1.81 ± 0.47	0.32 ± 0.01

**Reference strain **[13]	0.14 ± 0.02	0.17 ± 0.01

**CB2 strain *(pPMA1'p PGM2 *2 μ) **[13]	2.38	0.23 ± 0.02

Specific PGM activity was measured in extracts of cells grown in defined medium containing galactose (20 g l^-1^). Specific growth rates (μ_max _± standard deviation) were measured under aerobic conditions in defined medium with 50 g l^-1 ^galactose. For comparison, data reprocessed from Bro et al [13] are also reported.

**Figure 1:**
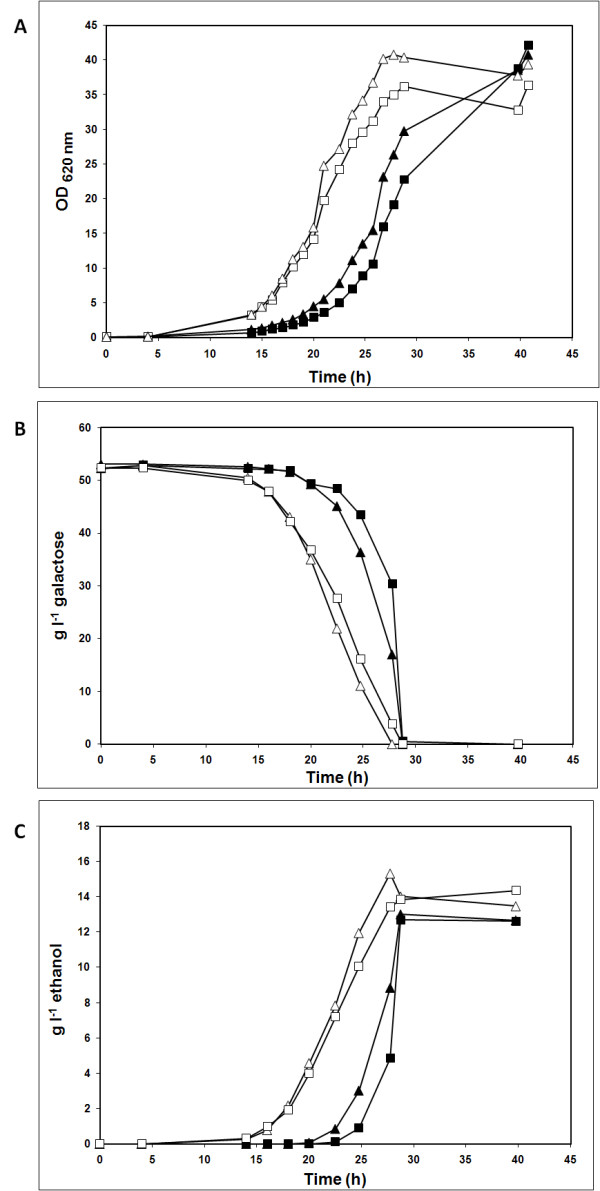
**Effect of PGM level on aerobic growth on galactose**. Aerobic batch with YNB medium supplemented with 50 g l^-1 ^galactose. Inocula pre-grown on YNB medium with 20 g l^-1 ^glucose. Experiments were performed in duplicate. (A) Biomass formation (OD _620 nm_). (B) Galactose consumption. (C) Ethanol production. *S. cerevisiae *strains: Control m (filled square), PGM2 m (open square), Control i (filled triangle) and PGM2 i (open triangle).

Next, the *PGM2 *overexpressing strains and their respective control strains were compared with respect to aerobic growth in galactose medium (Table 2, Figure 1). In aerobiosis on galactose, growth, galactose consumption and ethanol production were similar for the two control strains, **Control m **and **Control i **(Figure 1). Also the strains overexpressing *PGM2*, from a multicopy plasmid, **PGM2 m**, and from an integrated plasmid, **PGM2 i**, respectively, displayed similar results (Figure 1). The maximum specific growth rates (μ_max_) were 0.22 ± 0.01 and 0.24 ± 0.02 h^-1 ^for strains **Control m **and **Control i **respectively, whereas μ_max _for strains **PGM2 m **and **PGM2 i **increased to 0.30 ± 0.02 and 0.32 ± 0.01 h^-1^, respectively. Despite the fact that strain **PGM2 m **had 5 to 6 times higher PGM activity than strain **PGM2 i **when grown in galactose, μ_max _did not increase significantly. It was therefore decided to further investigate the influence of increased PGM activity only for strain **PGM2 i **and its control strain **Control i**. Additionally the fact that the extra-copy of *PGM2 *was stably integrated in the genome of the **PGM2 i **strain enabled us to study the effect of *PGM2 *overexpression independently of variations in plasmid copy number.

When the strains **Control i **and **PGM2 i **were grown aerobically in glucose medium μ_max _was 0.36 ± 0.01 h^-1 ^and 0.37 ± 0.04 h^-1^, respectively, which showed that the additional copy of the *PGM2 *gene did not affect growth on glucose. Assuming that the maximum specific growth rate on glucose (0.37 h^-1^) is also the theoretical maximum specific growth rate on galactose, overexpression of *PGM2 *results in a significant contribution to obtaining that growth rate.

### Anaerobic fermentation of galactose

**Figure 2:**
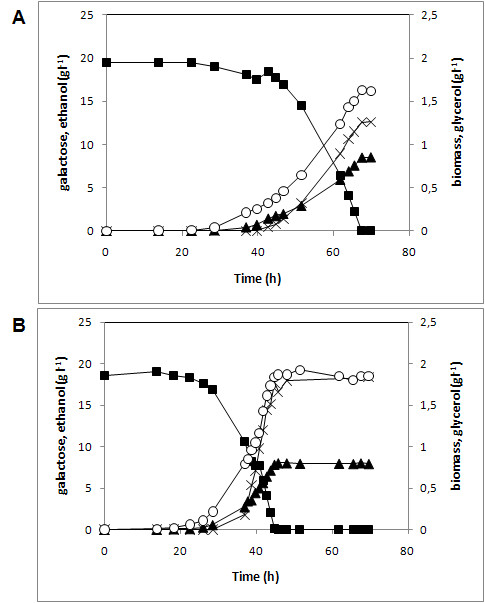
**Effect of *PGM2 *overexpression on anaerobic batch fermentation of galactose**. Sugar consumption and product formation in anaerobic batch fermentation of 20 g l^-1 ^galactose in defined medium with strains (A) Control i and (B) PGM2 i. Cells for inocula were pre-grown in defined medium with 20 g l^-1 ^glucose. Experiments were performed in duplicate. Symbols: galactose (filled square), ethanol (filled triangle), biomass (DW) (open circle), glycerol (cross symbol).

Anaerobic fermentation of galactose with the strains **Control i **and **PGM2 i**, showed that overexpressing *PGM2 *reduced the time required to deplete galactose with about 24 h ± 1 h (Figure 2A and 2B). The increased PGM activity consistently reduced the lag phase of fermentative galactose-grown cells (pre-cultivated on glucose) (Figure 2). This was accompanied by an increase of the maximum specific growth rate from 0.098 h^-1 ^± 0.003 to 0.139 h^-1 ^± 0.004 (p-value < 0.01) (Table 3). The ratio between the different products of galactose was much less influenced by the increased PGM activity. The ethanol yield was the same for strains **Control i **and **PGM2 i**. However, the reduced lag phase and the increased maximum specific growth rate translated into a shorter fermentation duration from 69.3 ± 0.8 h in the **Control i** strain to 45.5 ± 0.4 in the **PGM2 i **strain.

**Table 3:** Parameters analyzed during anaerobic batch fermentation of galactose.

Strain	**Maximum specific growth rate μ**_**max**_ (h^**-1**^) ^**a**^	Biomass yield (g g galactose ^**-1**^)	Glycerol yield (g g galactose^**-1**^)	Ethanol yield (g g galactose ^**-1**^)	Fermentation time (h)
**Control i**	0.098 ± 0.003	0.08 ± 0.00	0.07 ± 0.01	0.41 ±0.02	69.3 ± 0.8

**PGM2 i**	0.139 ± 0.004	0.09 ± 0.01	0.09 ± 0.01	0.41 ± 0.02	45.5 ± 0.4

^a^Maximum specific growth rate was calculated by linear regression of a logarithmic plot on the phase of constant and maximum values.

## Discussion

The present study demonstrates for the first time that the introduction in the chromosome of an additional copy of the gene *PGM2 *encoding Pgm2p under the control of a strong and constitutive promoter increased the maximum specific growth rate on galactose in anaerobic fermentation. In addition the lag phase of glucose-pregrown cells in anaerobic galactose medium was significantly reduced. The extra integrated copy of the gene *PGM2 *increased the PGM activity 5 to 6 times in galactose-grown cells. Further overexpression of *PGM2 *from a multicopy plasmid increased the PGM activity almost 32 times as compared to the control strain but this did not further increase aerobic growth on galactose compared with overexpression from an extra chromosomal copy.

Pgm2p catalyses the last step in the Leloir pathway [8] where glucose 1-phosphate (G1P) is reversibly converted to the glycolytic intermediate glucose 6-phosphate (G6P). Pgm2p is the major isoform of the PGM enzyme in *S. cerevisiae *accounting for 80 to 90% of the PGM activity [15,16]. Pgm2p was originally believed to be constitutively present in high levels [10,15]. However, it was later found that the exposure to galactose gives lower induction of Pgm2p activity compared to other structural *GAL *genes that are at least 1000-fold induced [10,16]. The fact that overexpression of *PGM2 *under a strong constitutive promoter enhanced growth on galactose indicates that PGM is a controlling enzyme in the Leloir pathway.

Overexpression of *PGM2 *has previously been shown to increase the galactose uptake rate and the specific growth rate under aerobic conditions [13]. In the current study we confirmed these results in another strain background. When compared with the results of Bro et al, higher specific growth rates on galactose were obtained for both the control strains and the PGM2 overexpressing strains under aerobiosis. However higher concentrations of galactose were used in the present study. Nevertheless, in both studies the aerobic specific growth rates on galactose increased by 33-36% when the overexpressing *PGM2 *gene was overexpressed. We also found that, in our strain background, the increased PGM activity brought the aerobic specific growth rate in 50 g l^-1 ^galactose medium, 0.32 h^-1^, close to that in 20 g l^-1 ^glucose medium, 0.37 h^-1^.

Absence of anaerobic growth on galactose has been frequently observed for *S. cerevisiae *[17-19] and it has recently been ascribed to low cellular energy charge [18]. In the present study, an increased PGM activity resulted in improved anaerobic galactose utilization, which indicated that the energy charge deficiency may not be the only limitation of the Leloir pathway. For instance PGM upregulation has been shown to increase the intracellular levels of glucose 6-phosphate [11,13,20] that is known to activate glycolysis [9,21,22].

## Conclusions

Chromosomal integration of an additional copy of the *PGM2 *gene was sufficient to increase the anaerobic growth rate with 42% and reduce the fermentation time by 34%. Chromosomal integration allows for the construction of stable industrial strains that are expected to decrease the ethanol production time. These results have practical implications for industrial ethanolic fermentation of raw materials containing galactose, such as raffinose from the sugar industry [5], whey from the dairy industry [6] and lignocellulose for fuel ethanol production [2-4].

## Methods

### Strains

*Escherichia coli *DH5α and JM101 (Life Technologies, Rockville, MD, USA) were used for cloning. Plasmids and *Saccharomyces cerevisiae *strains (Table 1) were stored at -80°C in 15% glycerol. Freshly streaked plates from frozen stocks were used to inoculate pre-cultures.

### Molecular biology techniques

Standard molecular biology techniques were used for cloning [23]. Restriction enzymes, T4-DNA ligase and Shrimp Alkaline Phosphatase (SAP) were obtained from Fermentas (Vilnius, Lithuania). Yeast genomic DNA was purified with Easy-DNA Kit (Invitrogen, Groningen, The Netherlands). *Escherichia coli *DH5α competent cells were prepared as described before [24] and transformed using the calcium chloride method [25]. Yeast was transformed with the lithium acetate method [26]. Plasmids were extracted from bacteria either with GeneJET TM (Fermentas, Vilnius, Lithuania) or with QIAGEN Mini Plasmid Purification kit (Qiagen GmbH, Hilden, Germany). The QIAquick^® ^kit was used for Gel Extraction of DNA fragments from agarose gel as well as for PCR purification of amplified DNA products. Taq-polymerase (Fermentas, Vilnius, Lithuania) was used for analytical PCR, while Pfu-polymerase (Fermentas, Vilnius, Lithuania) and Pwo-polymerase (Roche Diagnostics GmbH, Mannheim, Germany) were used for High-fidelity PCR. Abi-Prism Big-Dye cycle sequencing kit (Applied Biosystems, Weiterstadt, Germany) was used for DNA sequencing that was performed by BM labbet AB (Furulund, Sweden).

### Construction of strains expressing multiple copies of the *PGM2 *gene

The YEplacHXT vector [27] that enables strong constitutive expression under the yeast promoter *HXT7' *[14] was linearized between the *HXT7'*promoter and *PGK *terminator using *BamHI *and *PstI*. Transformation of *S. cerevisiae *CEN.PK 113-11C [28] with the cleaved vector YEplacHXT generated strain TMB 3126 (Table 1). In parallel, the *PGM2 *gene was amplified from genomic DNA from TMB 3400 (Table 1) [29] using primers with overhangs (in bold) homologous to the 3' region of the *HXT7'*promoter(5'**TTTTTTAATTTTAATCAAAAAAGGATCCCCGGGCTGCA**ATGTCATTTCAATTGAAACG-3') and the 5' region of the *PGK *terminator (5'**CCACCACCAGTAGAGACATGGGAGATCTAGAATTCCT**TTAAGTACGAACCGTTGG-3'). The amplified fragment and the linearized plasmid were introduced in CEN.PK 113-11C to form the plasmid YEplacHXT-PGM2 by recombination (Table 1) and generated strain TMB 3127 (Table 1). The histidine auxotrophy of strains TMB 3216 and TMB 3127 was complemented by transforming the strains with a linear fragment containing the *HIS3 *locus that was amplified from YDp-H plasmid (Berben, Dumont et al. 1991) and selecting for growth in defined mineral medium without supplementation. The resulting strains were named **Control m **and **PGM2 m**, respectively (Table 1).

Plasmids were rescued and transformed into *E. coli *DH5α for verification. Analytical PCR of recovered plasmids confirmed that extra copies of *PGM2 *under the HXT7p' were present in **PGM2 m **but not in the **Control m **strain.

### Genomic integration of *PGM2*

The *HIS3 *amplicon was first transformed into *S. cerevisiae *CEN.PK 113-11C and transformants were selected on defined mineral medium supplemented with uracil. The resulting strain TMB 3134 (Table 1) was used to construct the strains **Control i**, TMB 3135 and **PGM2 i**, TMB 3136 (Table 1) as follows. The purified plasmid YEplacHXT-PGM2 (Table 1) from strain **PGM2 m **was used as template to PCR amplify the amplicon *HXT7'p-PGM2-PGKt* using Pwo-polymerase and the following primers: *PGKSalI *(5' ATCTGTCGAC**GACATAGAAATATCGAATGG **3') with sequence homologous to *PGKt *(in bold) and *HXTSalI *(5' ATCTGTCGA**CAGGAACAATTTCGGGCC **3') with sequence homologous to HXT7'p (in bold). The PCR product *HXT7'p-PGM2-PGKt* and the integrative vector YIplac211 [30] were cleaved with restriction enzyme *SalI *and ligated. The ligation mixture was transformed into *E. coli *DH5α competent cells and transformants were selected on LB plates with 100 mg l^-1 ^ampicillin. PCR and restriction analyses were used to confirm the proper size of the resulting plasmid YIplac211 HXT-PGM2.

Purified plasmids YIplac211 and YIplac211 *HXT-PGM2* (Table 1) from *E. coli *were cleaved with *Eco*RV to target the *URA3 *locus in strain TMB 3134. The prototrophic strain **Control i **and **PGM2 i **were generated by integration of cleaved YIplac211 and YIplac211 *HXT-PGM2* (Table 1), respectively. Genomic integration of *HXT7'p-PGM2-PGKt* was verified by analytical PCR of genomic DNA.

### Aerobic cultivation conditions

*E. coli *was grown and selected on Luria-Bertani medium (LB) [23] with 100 mg l^-1 ^ampicillin.

For yeast strains 6.7 g l^-1 ^Yeast Nitrogen Base medium (YNB, Difco Laboratories-Becton, Dickinson and Co., Sparks, MD, USA) was supplemented either with 50 g l^-1 ^galactose or 20 g l^-1 ^glucose as sole carbon source to assess growth. YNB liquid medium was buffered with potassium hydrogen phthalate (10.21 g l^-1 ^phthalate, 2.1 g l^-1 ^KOH, pH 5.5) [31]. For growth on plates 20 g l^-1 ^glucose and 20 g agar l^-1 ^was used. The concentration of YNB was doubled when the sugar concentration was above 20 g l^-1 ^to avoid nutrient limitation. Pre-cultures grown in YNB with 20 g l^-1 ^glucose until mid-late exponential phase overnight in 50 ml tubes were used to inoculate batch cultures at OD _620 nm _0.1-0.2 in cotton-stoppered baffled 500 ml flasks. To ensure initial aerobic conditions, the growth media was only 10% of the flask volume. YNB medium was supplemented with 40 mg l^-1 ^histidine and 20 mg l^-1 ^uracil for auxotrophic strains. Cultivation was performed at 30°C and 180-200 rpm agitation (Gallenkamp INR-200, Leicester, UK) at least in duplicate.

### Anaerobic fermentation

For anaerobic fermentation and corresponding pre-cultures defined mineral medium [32] was used. The medium for anaerobic fermentation was supplemented with 0.4 g l^-1 ^Tween 80 and 0.01 g l^-1 ^ergosterol and 20 g l^-1 ^galactose. The pre-culture medium contained 20 g l^-1 ^glucose and was buffered with phthalate buffer (10.21 g l^-1 ^phthalate, 2.1 g l^-1 ^KOH, pH 5.5) [31] whereas the bioreactor pH was controlled at 5.5 with automatic addition of 3 M KOH.

A first pre-culture was grown until late exponential phase in 50 ml tubes. The culture was used to inoculate a second aerobic pre-culture in 1000 ml cotton-stoppered baffled shake flasks. Both pre-cultures were grown in media, constituting 10% of vessel volume to assure maximum aeration. Cultures were grown at 30°C (Gallenkamp INR-200, Leicester, UK) and 180-200 rpm. Cells from the second pre-culture were grown until late exponential phase, washed twice with sterile water, centrifuged at 5000 rpm for 10 min, and used to inoculate anaerobic batch cultures at OD _620 nm _of 0.1-0.2.

Anaerobic batch fermentation was performed in 3 l Applikon^® ^Bio Reactors (Applikon, Schiedam, The Netherlands) with a working volume of 1.5 l, at 30°C and 200 rpm stirring. Prior to inoculation the bioreactor was flushed with nitrogen gas containing less than 5 ppm O_2 _(AGA Gas, Sundbyberg, Sweden) from the bottom of the bioreactor at a flow rate of 0.2 l min^-1 ^controlled by a gas mass flow-meter (Bronkhorst, HI-TECH, Ruurlo, The Netherlands). Outlet carbon dioxide and oxygen were monitored by a Carbon Dioxide and Oxygen Monitor type 1308 (Brüel & Kjaer, Copenhagen, Denmark). Off-gas condensers were cooled to 4°C to minimize ethanol evaporation and a dissolved oxygen probe monitored 0% oxygen tension. Anaerobic fermentation experiments were performed in at least biological duplicates and metabolite measurements varied with less than 10%. Cultivation was performed both with and without nitrogen gas flushing. Flushing resulted in an increased lag phase but did not affect the outcome of the experiment. Physiological characterization of recombinant strains was only performed with the prototrophic strains.

### Enzymatic activity measurements

PGM activity was determined in crude extracts of cells grown on YNB medium containing 20 g l^-1 ^galactose. For every strain, at least three independent biological replicates were assayed with at least two independent enzymatic measurements. Cells were harvested in exponential phase, centrifuged at 5000 rpm for 5 min, washed twice with cold sterile water and permeabilized with Y-PER (Pierce, Rockford, IL, USA). The protein concentration was determined with Coomassie Protein Assay Reagent (Pierce, Rockford, IL, USA), using bovine serum albumin as standard. Phosphoglucomutase activity was determined at 30°C by monitoring NAPDH production at 340 nm [13] with a Ultrospec 2100 pro spectrophotometer (Amersham Biosciences, Uppsala, Sweden). The chemicals used to determine enzyme activity were purchased from Sigma-Aldrich (St. Louis, MO, USA).

### Determination of metabolites and maximum specific growth rate

Concentrations of galactose, glycerol, acetic acid and ethanol were determined by high-pressure liquid chromatrography (HPLC) (Waters, Milford, MA, USA or Beckman Instruments Fullerton, CA, USA) with an Aminex HPX-87H ion exchange column (Bio-Rad, Hercules, USA) at 45°C. The mobile phase was 5 mM H_2_SO_4 _at a flow rate of 0.6 ml min^-1^. Cell dry weight was determined in at least duplicate at different time points of the fermentation experiment by filtering 5 ml culture through a pre-weighed and microwaved dried hydrophilic polyethersulfone 0.45 μM filter (PALL, Life Sciences, Michigan, USA). The filters were dried at 350 W for 8 min in a microwave oven and cooled prior to weighing.

The maximum specific growth rate was calculated by linear regression on a logarithmic plot of the phase of constant and maximum values.

## Competing interests

The authors declare that they have no competing interests.

## Authors' contributions

RGS participated in the design of the study, performed the experimental work and drafted the manuscript. BHH and MFGG designed the study and edited the manuscript. All authors read and approved the final manuscript.
